# Case report: A safeguard in the sea of variants of uncertain significance: a case study on child with high risk neuroblastoma and acute myeloid leukemia

**DOI:** 10.3389/fonc.2023.1324013

**Published:** 2024-01-08

**Authors:** Francesco Fabozzi, Rosalba Carrozzo, Mariachiara Lodi, Angela Di Giannatale, Selene Cipri, Chiara Rosignoli, Isabella Giovannoni, Alessandra Stracuzzi, Teresa Rizza, Claudio Montante, Emanuele Agolini, Michela Di Nottia, Federica Galaverna, Giada Del Baldo, Francesco Del Bufalo, Angela Mastronuzzi, Maria Antonietta De Ioris

**Affiliations:** ^1^ Hematology/Oncology, Cell and Gene Therapy, IRCCS Bambino Gesù Children’s Hospital, Rome, Italy; ^2^ Unit of Cell Biology and Diagnosis of Mitochondrial Disorders, Laboratory of Medical Genetics, IRCCS Bambino Gesù Children’s Hospital, Rome, Italy; ^3^ Pathology Unit, IRCCS Bambino Gesù Children’s Hospital, Rome, Italy; ^4^ Laboratory of Medical Genetics, Translational Cytogenomics Research Unit, IRCCS Bambino Gesù Children’s Hospital, Rome, Italy; ^5^ Unit of Cell Biology and Diagnosis of Mitochondrial Disorders, Neuromuscular Disorders Research Unit, IRCCS Bambino Gesù Children’s Hospital, Rome, Italy

**Keywords:** cancer predisposition syndrome, succinate dehydrogenase complex subunit C (SHDC) gene, variants of uncertain significance (VUS), neuroblastoma, acute myeloid leukemia

## Abstract

The increased availability of genetic technologies has significantly improved the detection of novel germline variants conferring a predisposition to tumor development in patients with malignant disease. The identification of variants of uncertain significance (VUS) represents a challenge for the clinician, leading to difficulties in decision-making regarding medical management, the surveillance program, and genetic counseling. Moreover, it can generate confusion and anxiety for patients and their family members. Herein, we report a 5-year-old girl carrying a VUS in the Succinate Dehydrogenase Complex Subunit C (*SHDC)* gene who had been previously treated for high-risk neuroblastoma and subsequently followed by the development of secondary acute myeloid leukemia. In this context, we describe how functional studies can provide additional insight on gene function determining whether the variant interferes with normal protein function or stability.

## 1 Introduction

Secondary malignancies represent a major challenge in pediatric cancer patients with an incidence up to 20% among survivors (1). Myeloid neoplasms may occur from a few years to several decades after cancer treatment and they are more frequent in cancer-predisposing germline variants carriers. Therapy-related myeloid neoplasms (t-MNs) have an incidence of 0.62 per 100,000 and account for 10-20% of new diagnoses of acute myeloid leukemia/myelodysplastic syndrome in adulthood; there are few and controversial data available in the literature on t-MNs in pediatric age, although the most widely accepted hypothesis is that pathogenic germline variant (e.g., in the *NF1* gene) may interact with the genotoxic effect of chemotherapeutics, inducing the development of the secondary neoplasm (2, 3).

Although all cytotoxic drugs increase the risk of developing a myeloid neoplasm, the two classes of chemotherapeutics most associated with the development of t-MNs are alkylating agents and topoisomerase II inhibitors (2).

The advances in molecular biology and genetic technologies have significantly improved the detection of germline variants in cancer patients with important implications in the management and in the decision making (4). However, the identification of these variants is not a straightforward process and the interpretation of the results is often challenging. One of the most significant burdens is represented by the Variants of Uncertain Significance (VUSs) detection, with their uncertain role and impact on the pathogenesis of neoplasms. The presence of VUSs can lead to confusion and anxiety for the individual and their family members, as well as difficulties in decision making regarding the medical management, the surveillance program and the genetic counseling. In this context, functional studies can provide additional insights regarding the possible influence of the variant on the normal protein functions and its stability.

Herein we describe a case of acute myeloid leukemia (AML) arising in a 5-year-old girl previously treated for high-risk neuroblastoma (NB).

At the onset of the second malignancy, the patient underwent genetic testing with Next Generation Sequencing (NGS) technology to identify germline genetic variants predisposing to cancer. The study found a VUS in the Succinate Dehydrogenase Complex Subunit C (*SDHC)* gene. Therefore, it was decided to perform a molecular study on tumor tissue, which detected the presence of the same variant in the neuroblastoma cells of the primary tumor.

Finally, a functional study was performed on the patient’s fibroblasts to clarify the potential impact and causal role of this variant.

## 2 Case report

A 3-year-old-girl was diagnosed with high-risk neuroblastoma (NB). She underwent an intensive front-line chemotherapy treatment according to European guidelines (5), without achieving a complete remission (CR) at the end of the induction phase. She received a second-line treatment including four courses of Temozolomide-Irinotecan (TEMIRI) followed by radiometabolic therapy with high-dose ^131^I-Metaiodobenzylguanidine (MIBG) and melphalan supported by autologous stem cell transplantation (aSCT). Subsequently, she underwent high-dose chemotherapy (Busulfan and melphalan) with a further aSCT and chemoimmunotherapy with five courses of TEMIRI and Dinutuximab beta, surgery and radiotherapy on primary tumor (see **Supplementary Table 1**). This very intensive treatment resulted in a CR. Unfortunately, a bone marrow evaluation during early follow-up showed the onset of secondary AML carrying t(9;11) *MLL-AF9* translocation (**Supplementary Figure S1**).

**Figure 1:**
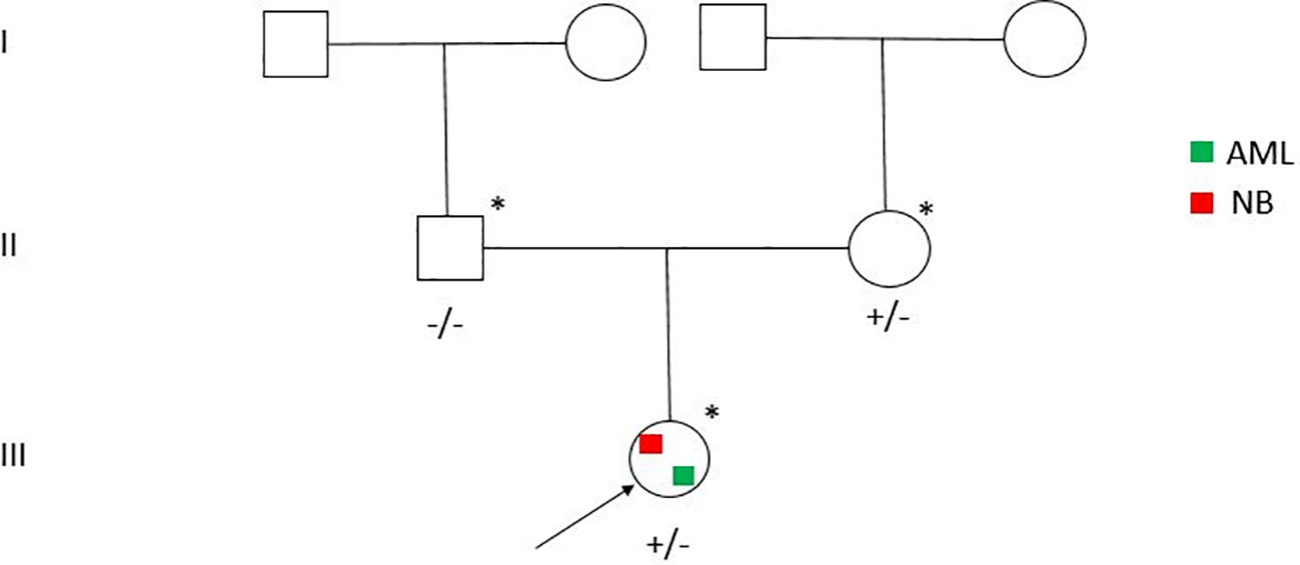
Pedigree of the family. Individuals with SDHC c.197C>T (p.Ala66Val) positive test are indicated by the plus sign.

Due to the early arising of a secondary leukemia, an NGS was performed using the Twist Custom Panel kit (clinical exome - Twist Bioscience) on NovaSeq6000 platform (Illumina), filtering for a dedicated diagnostic gene panel (ALK, APC, AXIN2, BARD1, BRCA1, BRCA2, BRIP1, CDKN1C, CHEK2, ERCC4, EZH2, FANCA, FANCB, FANCC, FANCD2, FANCE, FANCF, FANCG, FANCI, FANCL, FANCM, HRAS, KIF1B, KRAS, LZTR1, MAD2L2, NF1, PALB2, PHOX2B, PTPN11, RAF1, SDHAF2, SDHB, SDHC, SDHD, SLX4, SOS1, TP53, UBE2T, XRCC2) (6, 7). In addition, the analysis was extended filtering for a large cancer predisposition gene panel (8, 9) (**Supplementary Table 2**). The BaseSpace pipeline (Illumina, https://basespace.illumina.com/) and the GeneYX software (LifeMap Sciences) were used for the variant calling and annotating variants, respectively. Sequencing data were aligned to the hg19 human reference genome. Variants were examined for coverage and Qscore (minimum threshold of 30), and visualized by the Integrative Genome Viewer (IGV) (**Supplementary Table 3**). Exome sequencing revealed the missense variant c.197C>T (p.Ala66Val) (rs760572684) in *SDHC* (NM_003001.5) gene, in heterozygous state and maternally segregated (**Figure 1**), classified as VUS according to American College of Medical Genetics (ACMG) [BP4, PM1, PM2 criteria] (6). Conservation score (phyloP100) is -0.952. The variant is reported in GnomAD (exomes: *f* = 0.0000241; genomes: *f* = 0.0000637). The pathogenicity score is heterogeneous: meta score underlines five algorithms as uncertain prediction (BayesDel noAF, BayesDel_addAF, MetaSVM, MetaLR, REVEL) vs one benign prediction (MetaRNN). In addition, 12 individual prediction algorithms show a benign prediction (DANN, DEOGEN2, EIGEN, EIGEN PC, FATHMM-MKL, FATHMM-XF, LRT, M-CAP, MutationAssessor, PrimateAI, PROVEAN, SIFT) vs 4 uncertain (LIST-S2, Mutation Taster, MVP, SIFT4G) vs 1 pathogenic (FATHMM) (**Supplementary Table 4**). rs760572684 is annotated in ClinVar as a Variant of Uncertain Significance (VUS) in patients with Hereditary Cancer-Predisposing Syndrome [RCV001013940], Gastrointestinal Stromal Tumor [RCV000529986] and SDHC-Related Hereditary Paraganglioma-Pheochromocytoma Syndrome (Paragangliomas 3) [RCV000410077] and has been previously described (10). To note, the mother is in good health with mute clinical history.

In addition, the variant analysis, performed filtering on the extended gene panel, revealed a rare maternally inherited heterozygous missense variants, c.943T>C (p.Phe315Leu) in *PINK1* gene (NM_032409.3). This variant can be classified as VUS according to American College of Medical Genetics (ACMG) (PP3, PM1 and PM2). The *PINK1* gene is know to be associated with Parkinson disease (11) and was reported as a candidate NB susceptibility gene (12).

In view of the peculiar clinical history characterized by the occurrence of two nearly synchronous neoplasms (NB and AML) and the finding of the rs760572684, a NGS analysis on primitive tumor was performed. DNA was extracted from formalin-fixed paraffin-embedded primary tumor tissue using NucleoSpin Tissue Kit (Machery-Nagel) according to the manufacturer’s protocol, and NGS data were analyzed with Illumina TruSight Oncology 500. The molecular analysis found 5 VUSs: c.197C>T (p.A66V) on *SDHC* gene (the same variant found in germinal sample) (AF: 43.4%); c.1202G>A (p.R401K) on the *BIRC3* gene (AF: 55.7%); c.1012G>C (p.E338Q) and c.650_651delinsAA (p.P217Q) on the *EPHA3* gene (AF: 43.8% and 10.1%, respectively); and c.816A>C (p.K272N) on the *MAP3K1* gene (AF: 11%). Of note, no second-hit mutation was found in the other *SDHC* allele.

To further investigate the possibility of a causative role of mutated SDHC, a functional analysis was performed by 3 different tests; thus, the patient underwent skin biopsy in order to obtain fibroblasts for these studies.

**Figure 2:**
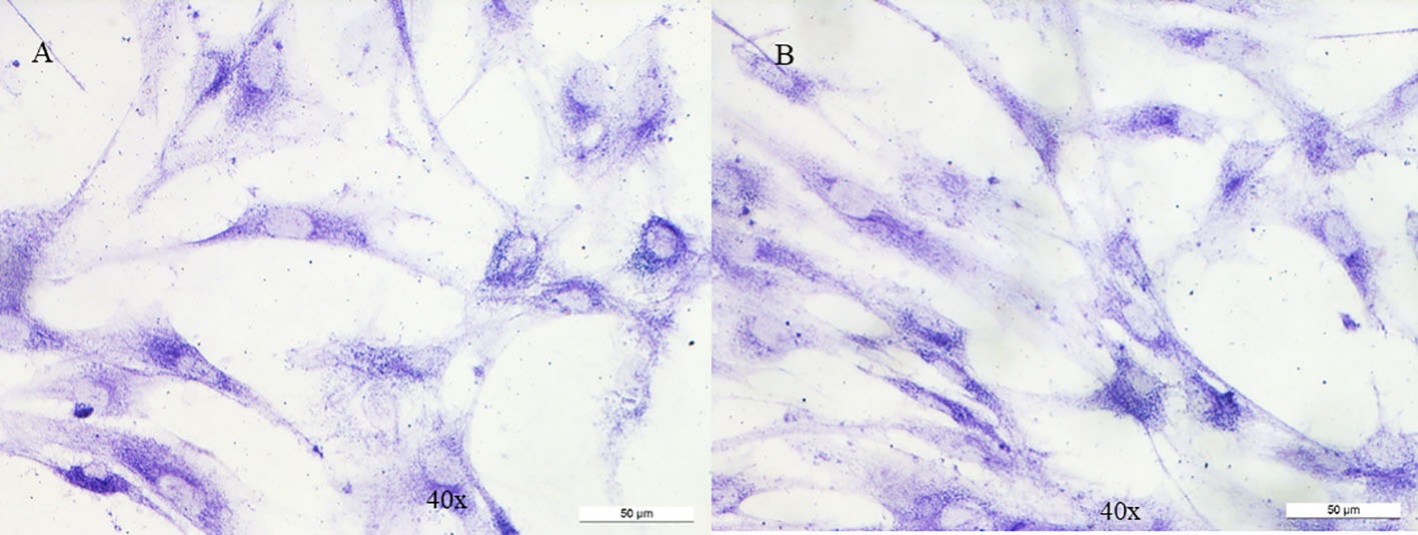
Cytochemistry for succinate dehydrogenase on fibroblasts. No variation is observed between control **(A)** and patient **(B)**.

**Figure 3:**
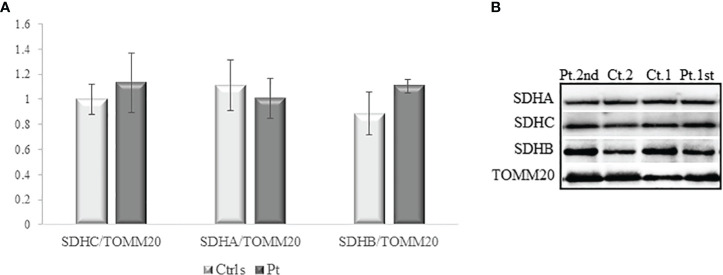
Western blotting on homogenate of fibroblasts. **(A)** Densitometric analysis of five experiments on three different fibroblast extractions for SDHC, SDHA and SDHB subunits. TOMM20 has been used for normalization of the referred proteins. **(B)** A representative experiment out of the five performed. No variation is observed between control and patient for the SDHC subunit, as well for SDHA and SDHB. Ct.1 and Ct.2: two different controls fibroblasts; Pr.1sr and Pt.2^nd^: two different protein extraction from patient’s fibroblasts.

Fibroblasts underwent a cytochemical investigation for the enzyme succinate dehydrogenase (**Figure 2**); through mitochondrial respiratory chain complex V enzyme activity, we evaluated adenosine triphosphate (ATP) production in mitochondria purified from fibroblasts using different substrates: succinate, specific for succinate dehydrogenase (respiratory chain complex II) and two others specific for NADH dehydrogenase (respiratory chain complex I) (**Figure 3A**); by western blotting we evaluated the content of the C subunit and 2 other subunits of succinate dehydrogenase (**Figure 3B**). The results showed that none of the three experiments detected an impaired function in the patient’s fibroblasts compared with the controls used, allowing the activation of BS3 criteria (well-established *in vitro* or *in vivo* functional studies show no damaging effect on protein function or splicing) of the American College of Medical Genetics (ACMG) guidelines (13).

## 3 Discussion

Among pediatric cancers, NB is the most frequent extracranial solid tumor of childhood (5). Whereas familial forms represent only 1–2% of all cases, whole-exome sequencing approaches have identified several rare germline variants that are associated with cancer susceptibility in patients with NB who lack the classic clinical criteria for a cancer predisposition syndrome (CPS). A comprehensive list of these genes is provided by Barr and Applebaum (6). It is usually inherited in an autosomal dominant manner, with incomplete penetrance, and like retinoblastoma, conforms to the classic two-hit model hypothesized by Knudson. Today, several predisposing genes for neuroblastoma are known, and, the first of which was identified in 2004 (8). *PHOX2B* gene at 4p12, a neurogenesis regulatory gene, was the first gene whose role in neuroblastoma predisposition was discovered, and its mutations have been found in up to 10% of familial, often multifocal, NBs as a part of neurocristopathy syndrome, also characterized by Hirschsprung disease and congenital central hypoventilation (14).

Germline mutations in the ALK gene have also been associated with hereditary neuroblastoma cases; *ALK* encodes for a receptor tyrosine kinase and it is frequently mutated in NB with unfavorable biologic features (15). Another gene that has recently been associated with the hereditary form of neuroblastoma is *MAX* (MYC Associated Factor X) gene, which encodes for an essential transcription factor in the MYC/MAX/MXD1 axis; specifically, germline mutations in *MAX* are associated with overexpression of *MYC*, a known unfavorable prognostic factor (16). Germline mutations in *TP53, SDHB, PTPN11, APC, NF1* and *NKAIN2* have also been reported to occur rarely in neuroblastoma patients and thus fall within the sequenced genes in the presence of clinical conditions suggestive of cancer predisposition (multiple tumor lesions, early age of onset, family history) (17–23). In addition to these genetic variants associated with NB predisposition, there are several CPS in which the risk of NB recurrence is higher and overlaps with the risk of other malignancies. In this case, the pathway of RAS-MAPK in diseases such as RASopathies plays a leading role (24).

We identified a missense variant c.197C>T (p.Ala66Val) (rs760572684) in *SDHC* (NM_003001.5) gene, in heterozygous state and maternally segregated in a 3-year-old girl with a metastatic NB diagnosis and AML diagnosis soon after the end of treatment for NB.

Weaver syndrome and familial paraganglioma/pheochromocytoma have also been linked to the development of neuroblastoma, with mutations found in *EZH2* and *SDHB* genes, respectively; the latter is a gene coding for one of the four subunits of the SDH complex involved in the mechanism of the electron transport chain and oxidative phosphorylation, which is essential for cellular ATP production (20, 25). Germline variants in the *SDHA*, *SDHB* and *SDHC* genes, encoding subunits of the SDH complex, have been associated with conditions characterized by the development of paragangliomas/pheochromocytomas, gastrointestinal stromal tumors and pulmonary chondromas (Carney triad and Carney-Stratakis syndrome) (26). In an article by Boikos et al., the presence of germline variants in *SDHx* genes in patients with Carney triad was investigated, concluding that in most cases the genetic defect remains unknown and that *SDHx* genes are apparently not involved in oncogenesis; moreover, although *SDHx* germline variants are present, the phenotype is often incomplete, suggesting the involvement of other genes, which may be contiguous with those encoding succinate dehydrogenase subunits (27).

**Figure 4:**
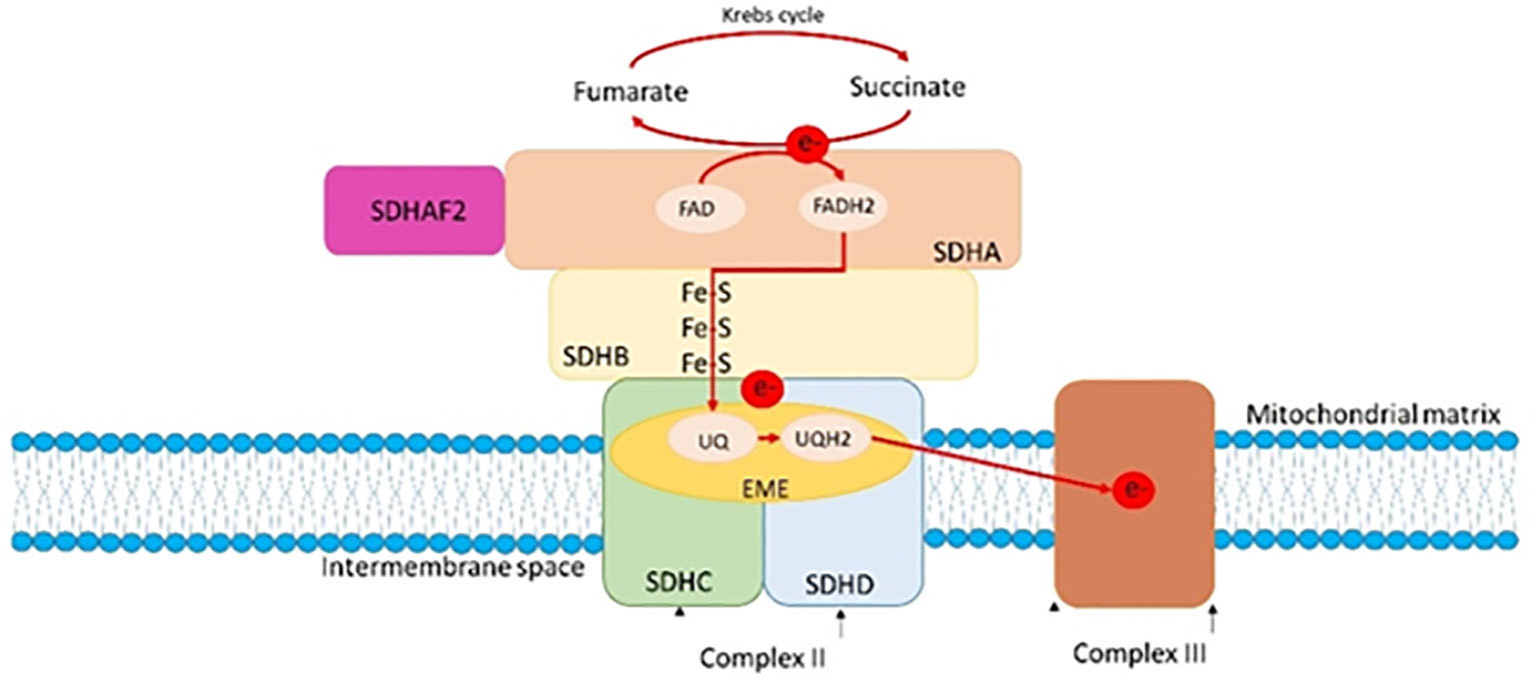
A: The SDH complex and its connection to the mitochondrial cristae and membranes. Taken together, the SDH subunits constitute the respiratory complex II. The hydrophilic SDHA and SDHB subunits catalyze the oxidation of succinate to fumarate as part of the tricarboxylic cycle, whereas the hydrophobic SDHD and SDHC subunits anchor the complex within the inner mitochondrial membrane. Prior to passing through Fe-S clusters in SDHB, tricarboxylic acid cycle (e-) electrons convert FAD to FADH2 in SDHA. These electrons then move on to the nearby respiratory complex III where they convert ubiquinone (Q) to ubiquinol (QH2).

The *SDHC* gene (Succinate Dehydrogenase Complex Subunit C) maps to the long arm of chromosome 1 (1q23.3) and it consists of six exons that code for a 169 amino acid (aa) polypeptide, which is one of the four subunits of succinate dehydrogenase (**Figure 4**) (28). Succinate dehydrogenase, also known as mitochondrial complex II, is a key heterotetrameric enzyme complex in the tricarboxylic acid cycle and aerobic respiratory chains of mitochondria. It consists of four subunits: two soluble subunits involved in electron transport, SDHA (primary catalytic subunit) and SDHB (redox subunit), and two transmembrane subunits, SDHC and SDHD, which anchor the complex in the inner mitochondrial membrane and facilitate electron transfer to the mitochondrial electron transport chain (29, 30). The oncological phenotype associated with *SDHx* variants is mainly due to loss of heterozygosity (LOH) events, although secondary somatic mutations are occasionally reported (31, 32). Defective activity of complex II leading to high oxygen (O_2_) production rate are detected in the cancer scenario, and in particular, several reports indicate that this defective activity may be caused by the pathogenic variants of the *SDHC* gene, that causes an increased O2 production and, consequently, an oxidative stress and genomic instability (33–35). In addition, genes involved in cellular respiration, as *SDHx* genes, act as tumor suppressors (30, 36, 37). *SDHC* variants are associated with gastrointestinal stromal tumors, paragangliomas and pheochromocytoma with an autosomal dominant transmission (31, 38–43). Insufficient data on the penetrance and phenotypic variability are reported in the literature, although some data show a lower penetrance in pathogenic variants of *SDHC* gene carriers compared with the *SDHD* pathogenic variants carriers (44–46).

Functional *in vivo* and *in vitro* studies showed that certain mutations in the *SDHC* gene inactivate the activity of the SDH complex in the yeast model and in the tumor tissue of patients, suggesting that these genes can act as tumor suppressor genes (47–49). In addition, studies on SDHC have shown that point mutations are associated with increased Reactive Oxygen Species (ROS) production, showing a correlation between dysfunction of all SDH complex subunits and formation of ROS (**Figure 4**) (50). In an elegant study by Ishii and colleagues, SDHC mutant transgenic cells showed elevated oxidative stress, increased transformation rate, tumor growth and DNA hypermutation in a mouse model (33). Another study, in which a nonsense mutation of *SDHC* was expressed in hamster fibroblasts, showed increased levels of ROS production, genomic instability and oxidative stress in mutant cells compared with parental cells (34). Finally, tumor formation and tumor cell proliferation may depend on the particular nature of the *SDHx* mutation (50).

Pathogenic and likely pathogenic variants of *SDHC* are splice site, frameshift and nonsense mutations, while there is a minority of missense variants (44). The variant found in our case (rs760572684) was described in the literature only in a *PTEN* mutation-negative patient, a 54-year-old patient with invasive breast cancer, follicular thyroid carcinoma, uterine leiomyomas, and cutaneous hemangioma with Cowden syndrome-like (10).

Of note, Trombetti and colleagues investigated the levels of *SDHC* variants and the oxidative mitochondrial metabolism in myeloid leukemia K562 cells over-expressing GATA-1 isoforms (51). They described a link between the levels of GATA-1S isoform (GATA Binding Protein 1 plays an important role in erythroid development by regulating the switch of fetal hemoglobin to adult hemoglobin) and *SDHC* alternative splicing variants (ASVs), leading to decreased of complex II activity and reduced oxidative phosphorylation efficiency, unveiling novel pro-leukemic mechanisms triggered by GATA-1S and aberrant expression of SDHC ASVs. Abnormal levels of SDHC and GATA-1S were also found in an AML patient, supporting *in vitro* results (51, 52). However, no *SDHC* germline mutations predisposing to AML have been described to date.

In our patient, no second hit mutation have been identified in the primary tumor. Furthermore, the functional study performed on the patient’s fibroblasts showed no variation between patient and control for cytochemistry analysis for succinate dehydrogenase, activity of the complex V of the mitochondrial respiratory chain on mitochondria isolated by fibroblasts and western blots on homogenate of fibroblasts, as well as *SDHC*, *SDHA* and *SDHB* are normal. Finally, the variant found in our patient involves a not conserved domain and we can assume that rs760572684 mutation might actually be a likely benign variant. Supporting this, the patient’s mother who presents the germline variant of the gene, is in good health and did not present history of diseases possibly related to the described mutation. Considering all, the second tumor may be more reasonably associated with iatrogenic effects of previous polichemotherapic treatment.

The case described highilights the importance of sharing new emerging gene mutations to improve the knowledge of molecular biology. Moreover, when available, the functional studies exploring the protein expression could help to define the role of VUS on the tumor history, in order to perform an appropriate follow-up and considering these mutations possible targets in a tailored therapy era. However, it should be emphasized that proband (or in this case parents) may decide to refuse to know the result of genetic testing.

## Data availability statement

The datasets presented in this study can be found in the National Center for Biotechnology Information. ClinVar; [VCV000371843.24], https://www.ncbi.nlm.nih.gov/clinvar/variation/VCV000371843.24 and National Center for Biotechnology Information. ClinVar; [VCV002663836.1], https://www.ncbi.nlm.nih.gov/clinvar/variation/VCV002663836.1, and can be found in the article/**Supplementary Material**.

## Ethics statement

Ethical approval was not required for the study involving humans in accordance with the local legislation and institutional requirements. Written informed consent to participate in this study was not required from the participants or the participants’ legal guardians/next of kin in accordance with the national legislation and the institutional requirements. Written informed consent was obtained from the individual(s) for the publication of any potentially identifiable images or data included in this article.

## Author contributions

FF: Writing – original draft. RC: Writing – original draft. ML: Writing – review & editing. AD: Writing – review & editing. SC: Writing – review & editing. CR: Writing – review & editing. IG: Writing – review & editing. AS: Writing – review & editing. TR: Writing – review & editing. CM: Writing – review & editing. EA: Writing – review & editing. MD: Writing – review & editing. FG: Writing – review & editing. GD: Writing – review & editing. FD: Writing – review & editing. AM: Writing – review & editing. MD: Writing – original draft.
