# Supplementary figures and images for: Case report: A safeguard in the sea of variants of uncertain significance: a case study on child with high risk neuroblastoma and acute myeloid leukemia
Source: Front Oncol. 2024 Jan 8;13:1324013. doi: 10.3389/fonc.2023.1324013 (PMC10800918; doi:10.3389/fonc.2023.1324013)

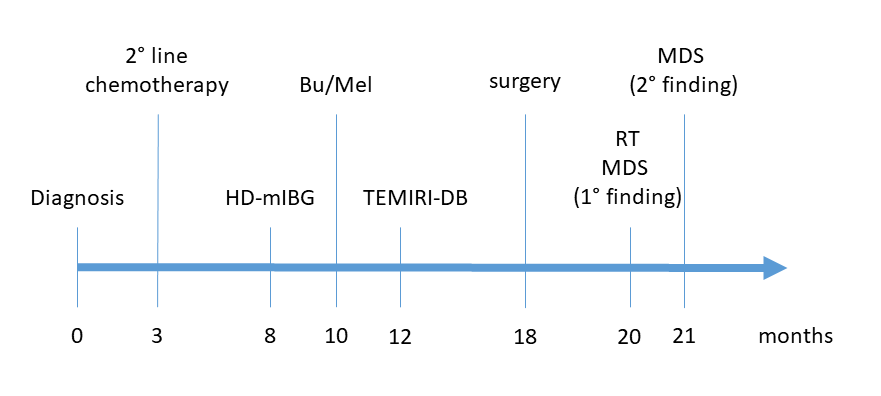

Supplement: Supplementary file 1 [file Image_1.tif]
