# Supplementary material for: Case report: A safeguard in the sea of variants of uncertain significance: a case study on child with high risk neuroblastoma and acute myeloid leukemia
Source: Front Oncol. 2024 Jan 8;13:1324013. doi: 10.3389/fonc.2023.1324013 (PMC10800918; doi:10.3389/fonc.2023.1324013)
Supplement: Supplementary file 2 [file Table_1.docx]

***Supplementary Material***

**A safeguard in the sea of variants of uncertain significance. A case study on child with high risk neuroblastoma and acute myeloid leukemia**

**Francesco Fabozzi^1*^, Rosalba Carrozzo^2^, Maria Chiara Lodi^1^, Angela Di Giannatale^1^, Selene Cipri^1^, Chiara Rosignoli^1^, Isabella Giovannoni^3^, Alessandra Stracuzzi^3^, Teresa Rizza^2^, Claudio Montante^1^, Emanuele Agolini^4^, Michela Di Nottia^5^**, **Federica Galaverna^1^, Giada Del Baldo^1^, Francesca del Bufalo^1^, Angela Mastronuzzi^1^, Maria Antonietta De Ioris^1^**

*** Correspondence:** francesco.fabozzi@opbg.net

**Supplemental Table S1**: Cumulative doses of cytotoxic drugs. WB (whole-body dose); BM (bone marrow dose)

| vincristine | 13,5 mg/mq |
| --- | --- |
| carboplatin | 1500 mg/mq |
| etoposide | 1400 mg/mq |
| cisplatin | 320 mg/mq |
| cyclophosphamide | 4200 mg/mq |
| topotecan | 15 mg/mq |
| adriamycin | 90 mg/mq |
| temozolomide | 2500 mg/mq |
| irinotecan | 2000 mg/mq |
| melphalan | 250 mg/mq |
| busulfan | 19,2 mg/mq |
| Dinutuximab-beta | 350 mg/mq |
| MIBG therapy dose | 3,6 Gy WB  1,8 Gy BM |
