# Supplementary material for: Case report: A safeguard in the sea of variants of uncertain significance: a case study on child with high risk neuroblastoma and acute myeloid leukemia
Source: Front Oncol. 2024 Jan 8;13:1324013. doi: 10.3389/fonc.2023.1324013 (PMC10800918; doi:10.3389/fonc.2023.1324013)
Supplement: Supplementary file 4 [file Table_3.docx]

***Supplementary Material***

**A safeguard in the sea of variants of uncertain significance. A case study on child with high risk neuroblastoma and acute myeloid leukemia**

**Francesco Fabozzi^1*^, Rosalba Carrozzo^2^, Maria Chiara Lodi^1^, Angela Di Giannatale^1^, Selene Cipri^1^, Chiara Rosignoli^1^, Isabella Giovannoni^3^, Alessandra Stracuzzi^3^, Teresa Rizza^2^, Claudio Montante^1^, Emanuele Agolini^4^, Michela Di Nottia^5^**, **Federica Galaverna^1^, Giada Del Baldo^1^, Francesca del Bufalo^1^, Angela Mastronuzzi^1^, Maria Antonietta De Ioris^1^**

*** Correspondence:** francesco.fabozzi@opbg.net

**Supplemental Table S3**: Targeted exome sequencing data output

| Targeted exome sequencing enrichment kit | Twist Custom Panel (Twist Bioscience) |
| --- | --- |
| Sequencing platform | Illumina NovaSeq 6000 |
| No. reads | 233,588,626 |
| Target regions coverage >20x | 99.27% |
| Average depth on target | 637.06 x |
| Total number of variants (passing variant quality filters) | 39,775 |
| Variants with effect on CDS or affecting canonical splice sites | 6,565 |
| Missense variants in the extended gene panel | 2 |
