# Supplementary material for: Case report: A safeguard in the sea of variants of uncertain significance: a case study on child with high risk neuroblastoma and acute myeloid leukemia
Source: Front Oncol. 2024 Jan 8;13:1324013. doi: 10.3389/fonc.2023.1324013 (PMC10800918; doi:10.3389/fonc.2023.1324013)
Supplement: Supplementary file 5 [file Table_4.docx]

***Supplementary Material***

**A safeguard in the sea of variants of uncertain significance. A case study on child with high risk neuroblastoma and acute myeloid leukemia**

**Francesco Fabozzi^1*^, Rosalba Carrozzo^2^, Maria Chiara Lodi^1^, Angela Di Giannatale^1^, Selene Cipri^1^, Chiara Rosignoli^1^, Isabella Giovannoni^3^, Alessandra Stracuzzi^3^, Teresa Rizza^2^, Claudio Montante^1^, Emanuele Agolini^4^, Michela Di Nottia^5^**, **Federica Galaverna^1^, Giada Del Baldo^1^, Francesca del Bufalo^1^, Angela Mastronuzzi^1^, Maria Antonietta De Ioris^1^**

*** Correspondence:** francesco.fabozzi@opbg.net

**Supplemental Table S4:** In-Silico Analysis Scores.

| **Metascores and individual predictions** | **score** |
| --- | --- |
| MetaRNN | 0.1866 |
| BayesDeladdAF | 0.09591 |
| BayesDel noAF | 0.06912 |
| MetaLR | 0.5472 |
| MetaSVM | -0.1875 |
| REVEL | 0.585 |
| SIFT | 0.674 |
| Mutation Taster | 0.9999 |
| DANN | 0.5751 |
| DEOGEN2 | 0.09456 |
| EIGEN | -1.1071 |
| EIGEN PC | -1.1515 |
| FATHMM-MKL | 0.00674 |
| FATHMM-XF | 0.05195 |
| LRT | 0.04033 |
| M-CAP | 0.02115 |
| MutationAssessor | 0.275 |
| PrimateAI | 0.3074 |
| PROVEAN | 0.38 |
| LIST-S2 | 0.8621 |
| MVP | 0.8017 |
| SIFT4G | 0.043 |
| FATHMM | -4.52 |
